# Ostéonécrose aseptique des condyles fémoraux révélée par une arthrite septique au cours de lupus érythémateux systémique

**DOI:** 10.11604/pamj.2015.22.94.7788

**Published:** 2015-10-01

**Authors:** Naziha Khammassi, Youssef Kort

**Affiliations:** 1 Faculté de médecine de Tunis,Service de Médecine Interne, Hôpital Razi, La Manouba 2010, Tunisie

**Keywords:** Lupus érythémateux systémique, ostéonécrose des condyles fémoraux, atteinte articulaire, SLE, osteonecrosis of the femoral condyles, joint involvement

## Image en medicine

L'ostéonécrose des condyles fémoraux n'est pas rare au cours du lupus érythémateux systémique (LES), elle peut prendre le masque d'une arthrite septique ou favoriser celle ci. L'IRM est l'examen de référence pour un diagnostic précoce. Le mécanisme physiopathologique reste controversé, l'hypothèse d'une vascularite des vaisseaux épiphysaires n'a pas trouvé de confirmation anatomique. Néanmoins, plusieurs facteurs de risque ont été rapportés (corticothérapie, anticorps antiphospholipides). Nous rapportons l'observation d'une patiente âgée de 37ans suivie depuis 1995 pour un LES compliqué d'un purpura vasculaire et d'un syndrome quadripyramidal ayant bien évolué après administration de boli de solumédrol relayés par une corticothérapie orale. Neuf ans après le début de la maladie, la patiente développe une impotence fonctionnelle du membre inférieur droit. L'examen clinique, le bilan biologique et radiologique ont conclu à une arthrite subaigüe du genou droit. Plusieurs diagnostics étaient évoqués (arthrite inflammatoire dans le cadre d'une poussée de sa maladie, une arthrite septique ou une simple poussée d'arthrose). L'arthrite septique est le premier diagnostic à éliminer car elle met en jeu le pronostic fonctionnel et vital, elle a bénéficié d'une arthrotomie parapatellaire, des soins locaux et une antibiothérapie. Néanmoins devant l'installation subaigüe de la symptomatologie et tout en sachant que l'arthrite septique au cours de LES, survient exceptionnellement sans point d'appel articulaire et elle complique volontiers une articulation fragilisée on a demandé une IRM du genou qui a objectivé une abrasion du cartilage et de l'os sous chondral des deux condyles fémoraux au niveau de leur versant postérieur (stade B de Mitchell).

**Figure 1:**
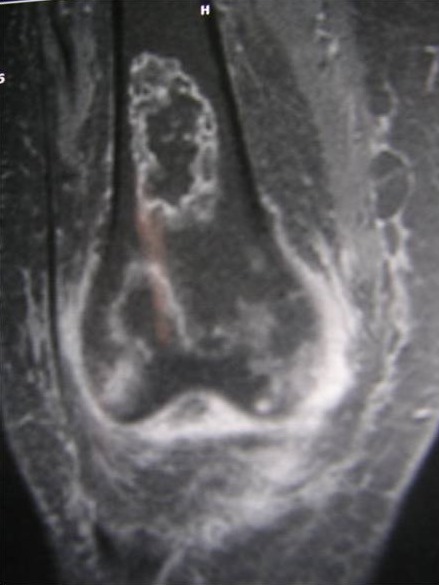
IRM du genou; ostéonécrose des condyles fémoraux

